# Supplementary figures and images for: Episome partitioning and symmetric cell divisions: Quantifying the role of random events in the persistence of HPV infections
Source: PLoS Comput Biol. 2021 Sep 7;17(9):e1009352. doi: 10.1371/journal.pcbi.1009352 (PMC8448377; doi:10.1371/journal.pcbi.1009352)

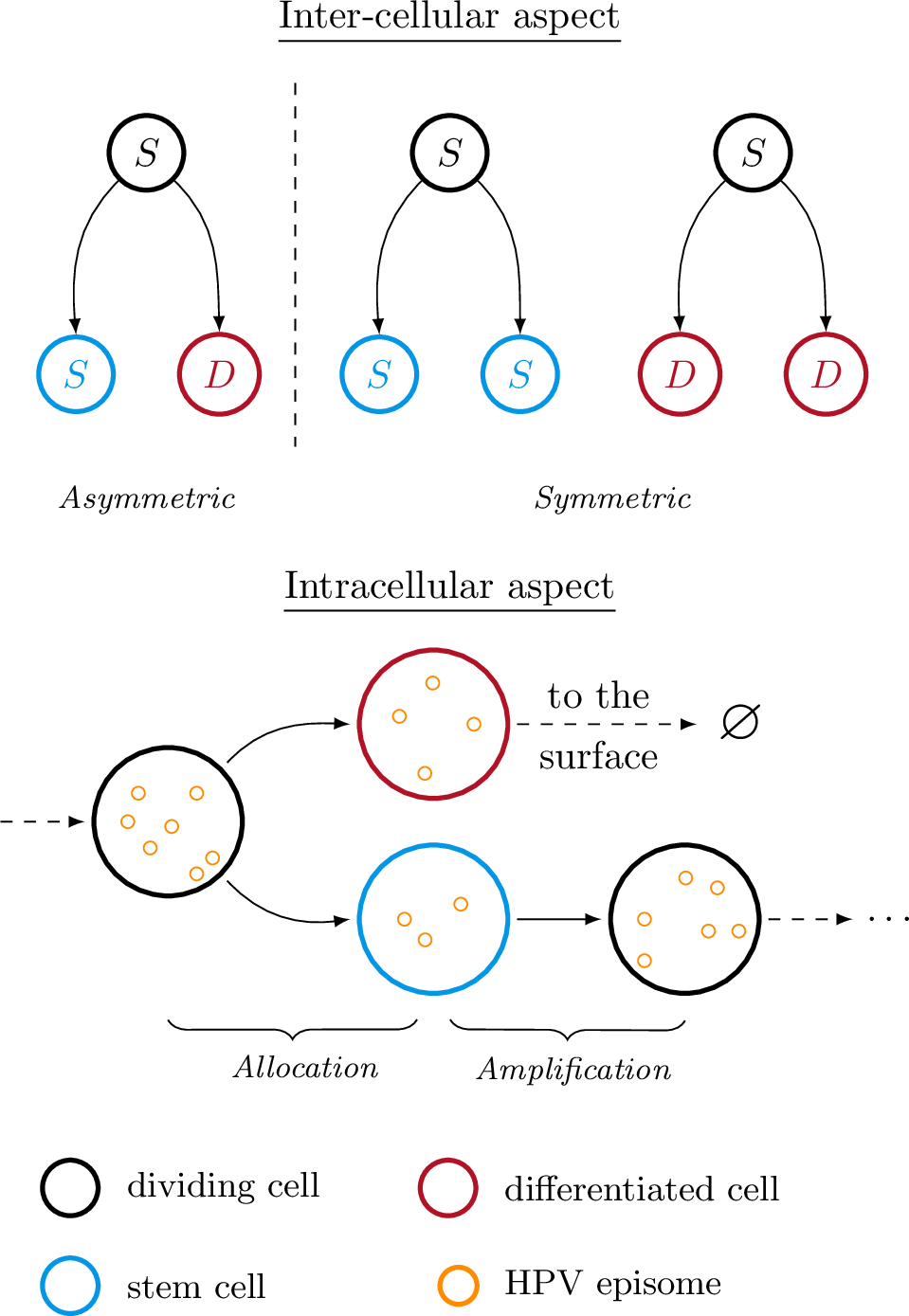

Supplement: S1 Fig — Stem cells in the basal layer divide mostly asymmetrically. Rare symmetric divisions occur that either yield two stem cells (with probability r) or two differentiated cells (with probability s). These can drastically impact the dynamic of the infection. Upon asymmetric division, viral copies are allocated randomly in the two daughter cells. Each episome is allocated independently to the daughter stem cell with probability p or else goes into the differentiated cell that migrates towards the epithelium surface. These cells do not participate in the persistence of the infection. Copies allocated to the stem cell are then amplified in λ copies on average. This random process is repeated every asymmetric division. The inter-cellular diagram is largely inspired from Fig 1 in reference [17]. (TIF) [file pcbi.1009352.s001.tif]

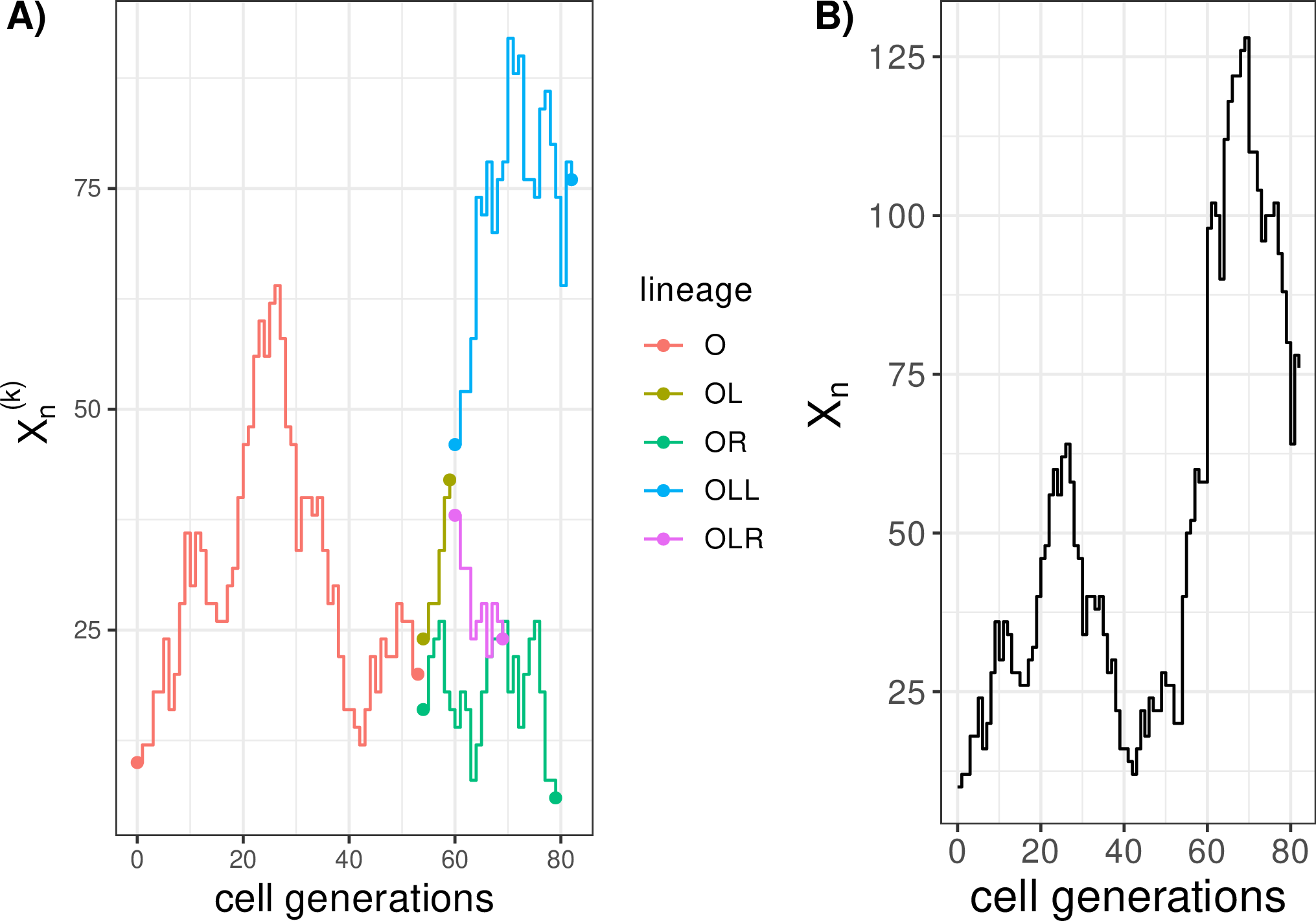

Supplement: S2 Fig — On the left panel (A) we displayed the dynamics inside each cell lineage over time. On the right panel (B) we plot the variation in the total number of episomes. We fixed the value of the parameters as follows: p = 0.5, λ = 2, s = r = 0.02, N0 = 10 and C = 200. The episomes amplification is fixed and happens after cell divisions. On panel (A), each color represents a cell lineage (more information on cell lineage labeling can be found in S1 Text). (TIF) [file pcbi.1009352.s002.tif]

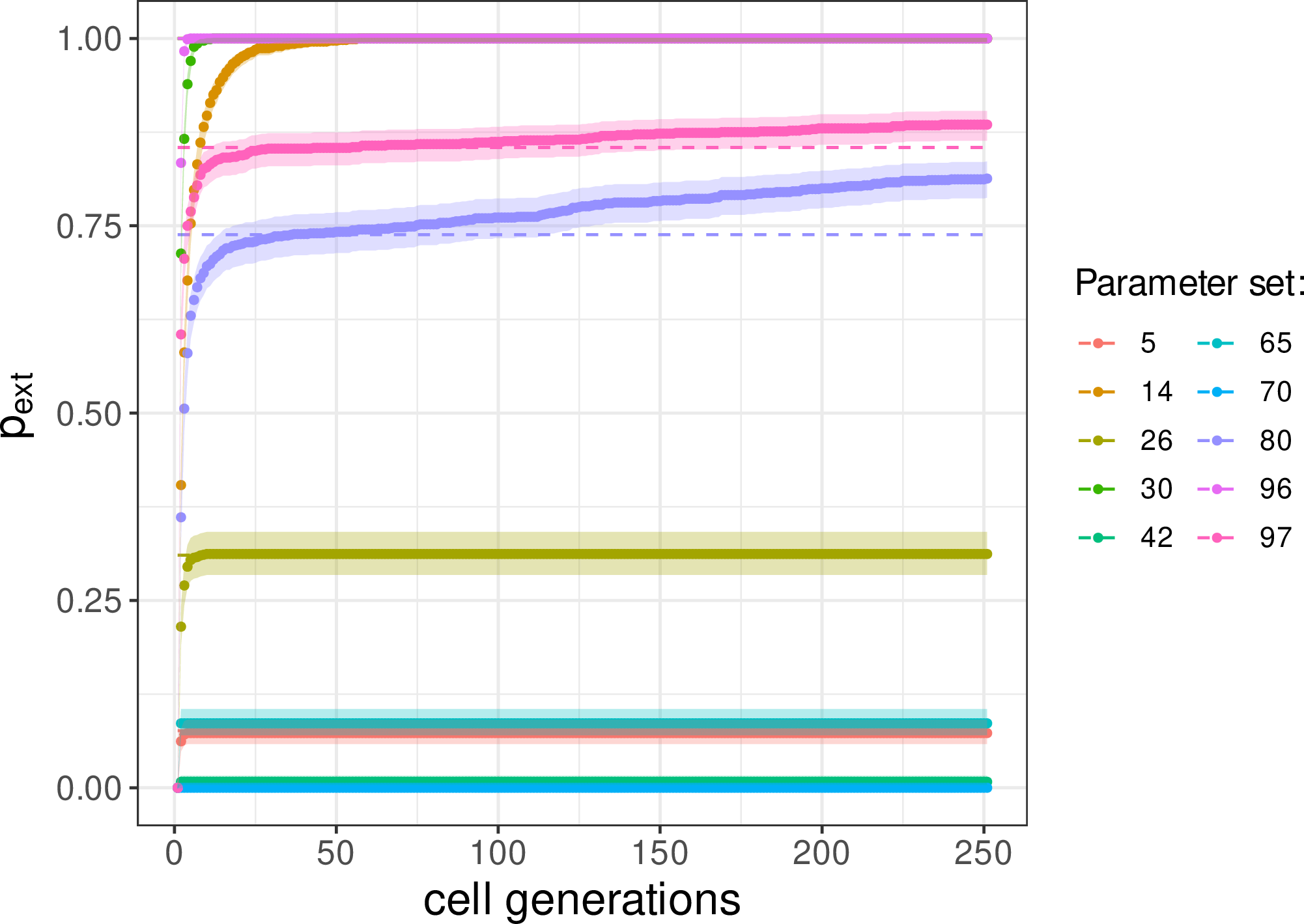

Supplement: S3 Fig — In most cases, the estimations of pext under constrained host capacity do not diverge from theoretical predictions on the time window considered. Deviations emerge when the intra-host regime is slightly supercritical and/or the intra-host capacity is limited (<50). The colors indicate the row of the parameter matrix LHS_intra (see S1 Text) used to obtain the cumulative probability of extinction. (TIF) [file pcbi.1009352.s003.tif]

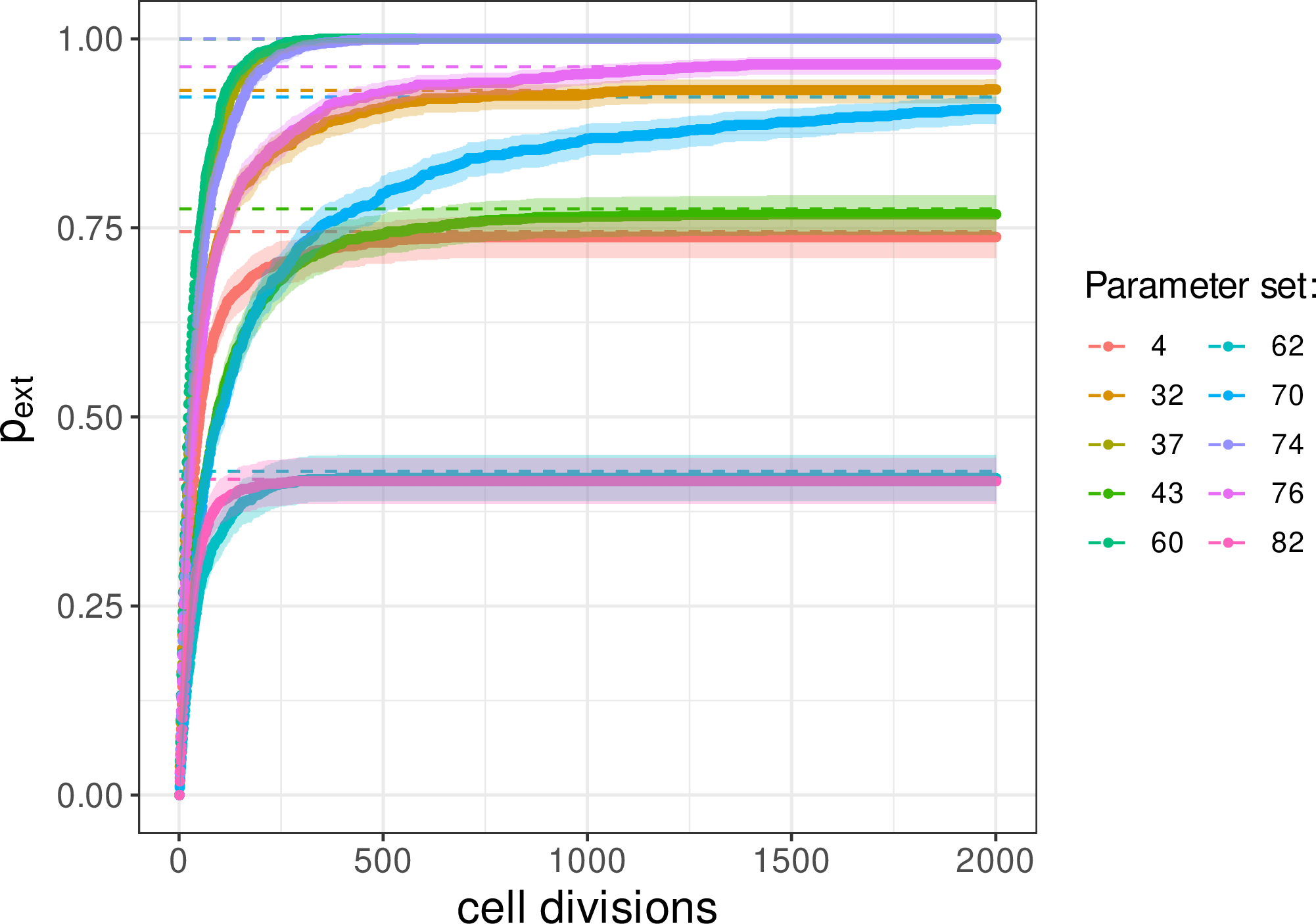

Supplement: S4 Fig — We observe no significant differences between estimations and theoretical predictions. The colors indicate the row of the parameter matrix LHS_inter (see S1 Text) used to obtain the cumulative probability of extinction. (TIF) [file pcbi.1009352.s004.tif]

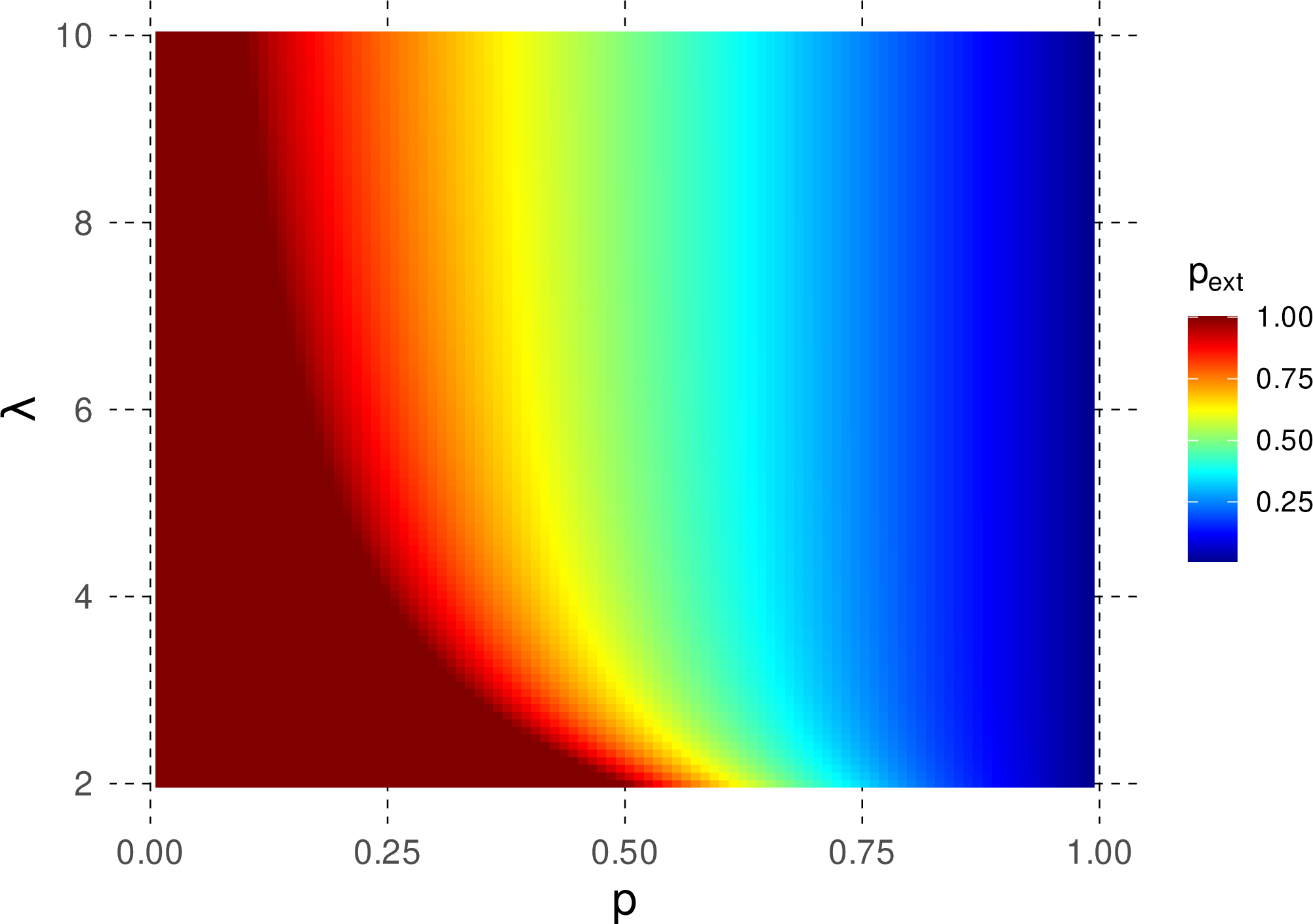

Supplement: S5 Fig — We here assume a Dirac scenario. pext decreases with both λ and p, and reaches a threshold pext = 1 − p for a given p when λ increases. This is consistent with the fact that if λ is sufficiently high, the main source of extinction is the first cell division of a stem cell containing 1 episome. (TIF) [file pcbi.1009352.s005.tif]

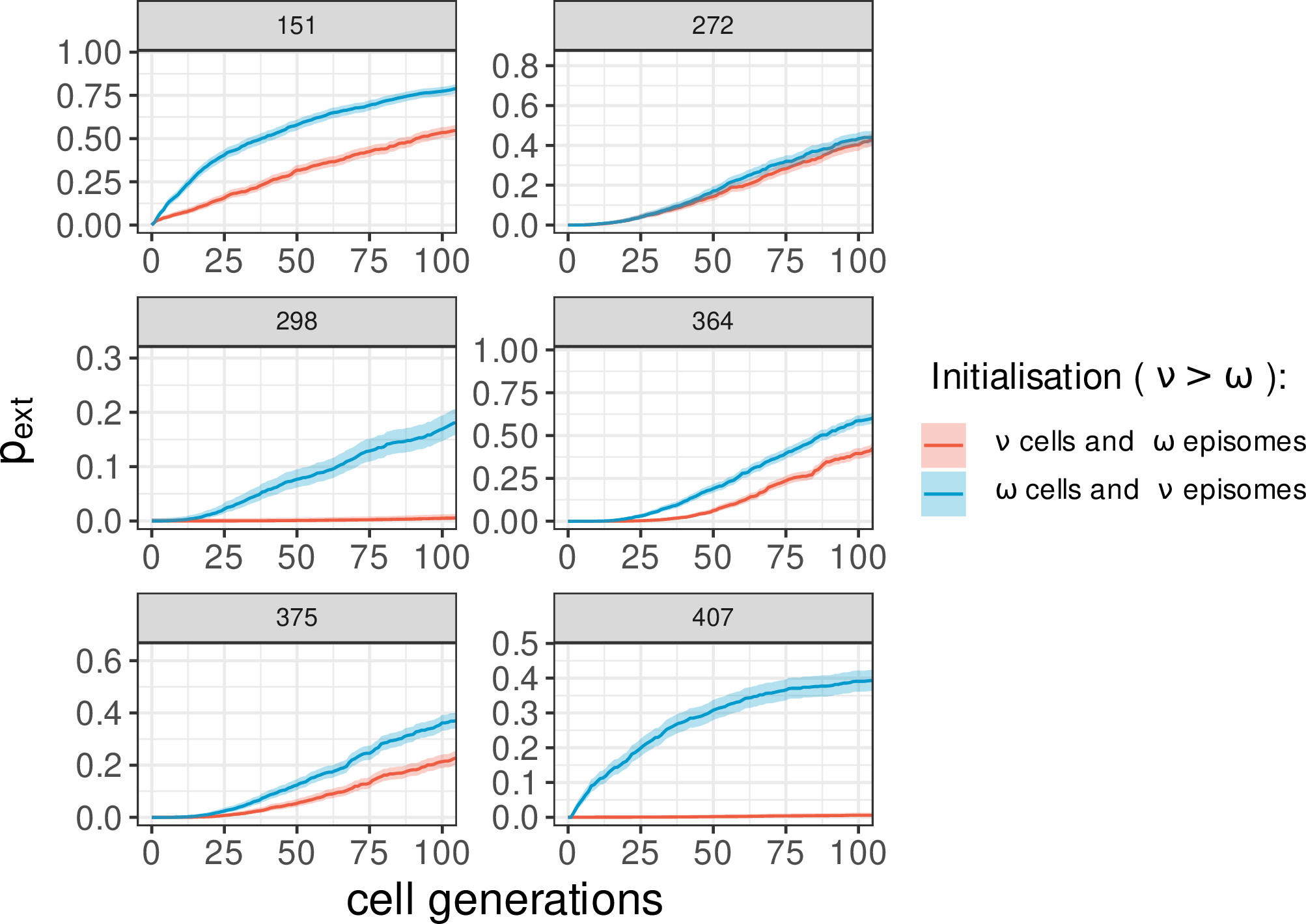

Supplement: S6 Fig — We display in red the estimations of pext in the scenario where the initial few viral copies are spread in more cells and in blue the scenario where more viral copies are spread in less cells. The solid lines indicates the estimations of pext and the ribbon the 95% confidence intervals. The cumulative probability of extinction is generally lower in the first scenario compared to the latter. Thus it is more beneficial for the virus to spread its copies in the maximum number of cells at the the start of the infection. On each facet, the title indicates the row of the parameter matrix LHS_A (see S1 Text). (TIF) [file pcbi.1009352.s006.tif]
