# Supplementary material for: Episome partitioning and symmetric cell divisions: Quantifying the role of random events in the persistence of HPV infections
Source: PLoS Comput Biol. 2021 Sep 7;17(9):e1009352. doi: 10.1371/journal.pcbi.1009352 (PMC8448377; doi:10.1371/journal.pcbi.1009352)
Supplement: S1 Text — (ZIP) [file pcbi.1009352.s007.zip › S1_Text.pdf]

Episome partitioning and symmetric cell divisions: quantifying the role of random events in the persistence of HPV infections

Thomas Beneteau *, Christian Selinger ◕, Mircea T. Sofonea ◕, Samuel Alizon ◕

MIVEGEC, Univ Montpellier, CNRS, IRD, Montpellier, France

◕These authors contributed equally to this work.
*Corresponding author: thomas.beneteau@ird.fr

Supporting Information

Details on intracellular branching processes

We distinguish two cases, constant or Poisson amplification, for the two processes are slightly different. We remind that X_n is a random variable following the total number of episomes in all infected stem cells. Conditioned on the absence of symmetric cell divisions, we model the variation of X_n over time as a Bienaymé-Galton-Watson (BGW) branching process defined by the following recurrence relation, for all $n \in \mathbb{N}$

$$X_{n+1} = \sum_{i=1}^{X_n} \xi_i \tag{1}$$

where $\xi_i = \xi$ are independent and identically distributed random variables modeling the offspring number of a single individual within a generation.

Dirac episome multiplication In this case, each episomes distributed to the daughter stem cell gives exactly λ new episomes then dies, so the random variable ξ can be written as

$$\xi = \begin{cases} 0 & \text{with probability } 1-p \\ \lambda & \text{with probability } p \end{cases} \tag{2}$$

Hence we deduce the associated probability generating function (PGF) $g(z), z \in [0,1]$:

$$g(z) = \sum_{k=0}^{\infty} \mathbb{P}[\xi = k]\, z^k = 1 - p + p\, z^{\lambda} \tag{3}$$

Following classical results on BGW branching processes [1], $\mathbb{P}[X_\infty = 0 | X_0 = 1] = p_{\text{ext}}$ is the smallest fixed point of the PGF on the interval $z \in [0,1]$:

$$p_{\text{ext}} = \inf\{z \in [0,1], g(z) = z\}$$

One can show that if $m = g'(1) = \lambda\, p \leqslant 1$, the average number of viral copies produced by one episome between each iteration, then $p_{\text{ext}} = 1$. If $m > 1$ one must solve the following polynomial equation to find p_{ext}

$$1 - p - z + pz^{\lambda} = 0 \tag{4}$$

For $\lambda \in \{2, 3\}$, such solutions are trivial but for higher value of λ, the solution becomes intractable. The S5 Fig shows the variation of extinction probability with the parameters λ and p.

Poisson episome multiplication We now assume that upon division, each episome dies and gives ρ descendants, where ρ is a random variable following a Poisson distribution, $Poisson(\lambda)$. Hence the distribution of ξ follows:

$$\mathbb{P}[\xi = k] = \mathbb{1}_{\{k=0\}}(1 - p) + p\,\mathcal{P}^{\lambda}(k) \tag{5}$$

where $\mathcal{P}^{\lambda}(k)$ is the probability mass function of the Poisson distribution $Poisson(\lambda)$. The PGF associated with such process is

$$g(z) = 1 - p + p\,e^{\lambda(z-1)} \tag{6}$$

Similarly to the previous case, if $m = \lambda\,p \leqslant 1$, the probability of extinction is equal to 1, else this probability is lower than 1. Let W be the Lambert function defined as the inverse function of $z \mapsto ze^{z}$. If $m > 1$ we have

$$p_{\text{ext}} = \frac{-W(-\lambda\,p\,e^{-\lambda\,p}) + \lambda\,(1 - p)}{\lambda} \tag{7}$$

The sensitivity of p_{ext} with the parameters λ and p are displayed in figure 2. Between the two cases, there are few differences, except for the low value of λ where $\mathcal{P}^{\lambda}(0)$ is non-negligible, hence favoring extinction compared to the Dirac case. We numerically checked our model converges to theoretical predictions. We display some results in S3 Fig. For the majority of the sets, the probability of extinction in our model does converge to numerical predictions. For some values, our model show higher values of p_{ext}, this notably happens when the intrahost capacity (C) and/or the regime is slightly supercritical. Theoretical predictions rely on the assumption the process might reach infinity. We diverge from this assumption when we constrain the intrahost capacity. Such constrain assure an asymptomatic probability of extinction equal to 1 even if the regime is supercritical. Yet, the time to extinction is increasing rapidly with C. Hence on our time interval of 3 years, in most cases, this phenomenon plays a negligible role.

Intracellular dynamics: amplification step prior to distribution step

Assume we reverse the two steps of the intracellular described in the "Methods"subsection. Upon division, the episomes are first amplified before being distributed in the two daughter cells. If we put the time origin just after the first amplification phase, we notice we are back in the scenario where the distribution of the viral copies occurs before the amplification phase. Therefore we deduce that a process starting with the amplification phase is equivalent to the same process starting with the distribution step and extra initial viral copies. From classical results on BGW branching process, we have the following results, for all $\nu \in \mathbb{N}^*$:

$$\mathbb{P}[X_\infty = 0|X_0 = \nu] = (\mathbb{P}[X_\infty = 0|X_0 = 1])^\nu \tag{8}$$

Let p_{ext}^D be the probability of extinction when the intracellular process starts with the distribution step and p_{ext}^A the probability of extinction when it begins with the amplification phase. For all $\lambda \in \mathbb{N}$ we hence have:

$$\left(p_{\text{ext}}^D\right)^\lambda = p_{\text{ext}}^A \tag{9}$$

Detail on the probability of extinction in the symmetric division case.

We remind the inter-cellular process is modeled using the following discrete BGW branching process. Let Γ_n be the random variable describing the total number of infected stem cells at time $n \in \mathbb{N}$. We note by $\widetilde{\Gamma}_n$ the random variable describing the dynamic of infected stem cells when the number of episomes in each stem cell is equal to ∞ (i.e. when omitting intracellular stochasticity). This variable is defined by the following recurrence relation:

$$\widetilde{\Gamma}_{n+1} = \sum_{i=1}^{\widetilde{\Gamma}_n} \phi_i \tag{10}$$

where $\phi_i = \phi$ are independent and identically distributed random variables modeling the offspring number of a single infected cell within a generation. From equation 2 we can characterized ϕ as follow

$$\phi = \begin{cases} 2 & \text{with probability } s \\ 0 & \text{with probability } r \\ 1 & \text{with probability } 1 - r - s \end{cases} \tag{11}$$

For all $z \in [0, 1]$, the PGF $h(z)$ associated to this reproduction law is defined as:

$$h : z \to r + (1 - r - s)\, z + s\, z^2 \tag{12}$$

We deduced that the average number of stem cell produced by each dividing stem cell is equal to $m = 1 - r + s$. The probability of extinction p_{ext} follows

$$p_{\text{ext}} = \inf\{z \in [0, 1], h(z) = z\} \tag{13}$$

In this case the results is straightforward and p_{ext} is the smaller roots of the polynomial equation

$$r - (r + s)\, z + s\, z^2 = 0,\; z \in [0, 1] \tag{14}$$

Hence we deduced $p_{\text{ext}} = \min\{\frac{r}{s}, 1\}$. We checked numerically our model converges the theoretical predictions and displayed some results in S4 Fig.

Lower bound of the probability of extinction

We remind that Γ_n (resp. $\widetilde{\Gamma}_n$) is the random variable describing the dynamic of infected stem cells over time in the general framework (resp. in the absence of intracellular stochasticity) at time n and $X_n^{(k)}$ the random variable characterizing the number of viral copies in lineage k at time n. We can write that:

$$\mathbb{P}[\Gamma_{n+1} = 0|\Gamma_n = \alpha] = \mathbb{P}[\widetilde{\Gamma}_{n+1} = 0|\widetilde{\Gamma}_n = \alpha] + \tag{15}$$

$$\sum_{i=1}^{\alpha} \mathbb{P}[\widetilde{\Gamma}_{n+1} = i|\widetilde{\Gamma}_n = \alpha] \times \sum_{k=1}^{i} \mathbb{P}[X_{n+1}^{(k)} = 0|X_n^{(k)} > 0] \tag{16}$$

$$\mathbb{P}[\Gamma_{n+1} = 0|\Gamma_n = \alpha] \geqslant \mathbb{P}[\widetilde{\Gamma}_{n+1} = 0|\widetilde{\Gamma}_n = \alpha] \tag{17}$$

This relation holds for all $n \in \mathbb{N}$, thus we deduce that

$$\mathbb{P}[X_\infty = 0|\Gamma_0 = 1] \geqslant \min\left(\frac{r}{s}, 1\right) \tag{18}$$

.

Parameters generations using LHS

We generate two matrices of parameters using R package "LHS" [2]. This package generates matrices $M = (m_{i,j})_{(1 \leqslant i \leqslant n, 1 \leqslant j \leqslant k)}$ where

$$0 \leqslant m_{i,j} \leqslant 1 \quad ; \quad 1 \leqslant i \leqslant n, 1 \leqslant j \leqslant k \tag{19}$$

As most of our parameters are defined outside of $I = [0, 1]$, we modify $(m_{i,j]})$ so that we get meaningful results regarding the parameter support. Let $[a_j, b_j]$ be the definition domain of variable j, $a_j \leqslant b_j$, then the value of parameter j for the i-th set, $S_{i,j}$ is

$$S_{i,j} = a_j + m_{i,j}(b_j - a_j) \tag{20}$$

When variable j is an integer we rounded up $S_{i,j}$. For the categorical variable describing the intracellular amplification regime (Dirac or Poisson distributed), we split the sampling space in two half: we assign the Fixed multiplication regime when $(m_{i,j}) < 0.5$ and the Poisson regime otherwise. See Table 1 for more details on the domain of definition of numerical variables. Additionally we set the upper bound for λ and N_0 to 10.

Annotation of infected cell lineages

Similarly to phylogeny, our process viewed from the infected stem cell perspective, can be visualized as a tree whose origin is the first infected cell. As long as the cells descending from that first infected cell divide asymmetrically, we remain on the same branch: we group such cells in a cell lineage. The moment, a stem cell divides symmetrically in two stem cells, the tree is split into two new branches that follow the same process independently. When a symmetric divisions into two differentiated cells occurs, we stop the branch. To track each branch we use the following notations.

i) The first cell lineage is labeled **O**.

ii) The two daughter stem cells are labeled **L** and **R** from its parent cell point of view. To distinguish them from other stem cells in the tree, they are named as follow:

- Take the parent's name (usually the letter **O** followed by a succession of **L** or **R**).
- Add the letter **L** or **R** to the parent's name.

For instance the two new stem cells lineage descending from the initial infected stem cell lineage are labeled **OL** and **OR**. Or, upon division, the stem cell lineage **OLLR** gives two new stem cell lineages **OLLRL** and **OLLRR**. The choice of **L** or **R** is random and have no influence on the rest of the process.

Simulation of our process

As the intracellular branching process is nested inside the inter-cellular branching process, we first need to compute the underlying tree of infected cells, then simulate the trajectories of the viral copies for each cell lineage (i.e. each branch) starting from the origin. We simulated the tree and the different trajectories using Python v3.9.1+.

Determination of the inter-cellular tree

The branching process for the inter-cellular dynamic can be viewed as a discrete birth-death process. Each individual, upon division, randomly takes one of the following three paths: i) divide symmetrically in two differentiated cells (death) with probability r ii) divide symmetrically in two stem cells (birth) with probability s iii) divide asymmetrically (pursue its life) with probability $1 - r - s$. Thus for each individual we can compute the time of symmetric divisions as it follows geometric distribution $Geometric(r + s)$. Once the time of symmetric division is determined we randomly assign of the two outcomes by drawing a random number from uniform distribution $Uniform([0, 1])$: if this number is lower than $r/(r + s)$ then the division gives two differentiated cells, otherwise the cell divides into two stem cells. We repeat this method until all cells have divided into two differentiated cells or when the remaining cell divisions time occur after a given threshold.

Intracellular dynamic for each branch

The intracellular branching process can also be consider as a Markov-chain. To simulate the dynamic of a Markov-chain we rely on its associated stochastic Matrix $P = (P_{i,j})_{(i,j)\in\mathbb{N}^2}$, where $P_{i,j} = \mathbb{P}[X_1 = j|X_0 = i]$. Depending on the episomes amplification regime (Fixed of Poisson - amplification or allocation phase first), the matrix varies. We detail the matrix P for the Fixed and Poisson cases when allocation occurs before amplification. We remind that when amplification occurs before allocation, the results on the probabilities of extinction are similar to the result obtained when the two phases are reversed raised to the power λ. First we explicit the stochastic matrix in the Fixed case:

$$P_{i,j} = \begin{cases} \mathcal{B}_p^i(j/\lambda) & \text{if } j \in \lambda\mathbb{N} \ ; \ j \leqslant \lambda\, i < C \\ 1 - \sum_{k=0}^{C-1} P_{i,k} & \text{if } j = C \\ 0 & \text{otherwise} \end{cases} \tag{21}$$

We note by $\mathcal{B}_p^n(x)$ the probability of drawing x individuals from a binomial distribution $Binomial(n, p)$. If we now assume a Poissonian amplification regime with distribution $Poisson(\lambda)$, then for all $(i, j) \in \mathbb{N}^2$:

$$P_{i,j} = \begin{cases} \sum_{k=0}^{i} \mathcal{B}_p^i(k)\,\mathcal{P}^{\lambda k}(j) & \text{if} \quad j < C \\ 1 - \sum_{k=0}^{C-1} P_{i,k} & \text{if} \quad j = C \\ 0 & \text{otherwise} \end{cases} \tag{22}$$

We note by $\mathcal{P}^n(k)$ the probability of drawing k individuals from a Poisson distribution $Poisson(n)$. As the state 0 is included, P is squared matrix of size $C+1 \times C+1$. Given the stochastic matrix P for all iteration $n \in \mathbb{N}$ and $i \in [\![0, C]\!]$, we iteratively determine the trajectory between two events of symmetric divisions by randomly choosing the next value for iteration $n+1$. We display an example of a random trajectory in S2 Fig.

References

1. Athreya KB, Ney PE. Branching Processes. Springer Berlin Heidelberg; 1972.
2. Carnell R. lhs: Latin Hypercube Samples; 2020. Available from: `https://CRAN.R-project.org/package=lhs`.
