# Supplementary material for: Episome partitioning and symmetric cell divisions: Quantifying the role of random events in the persistence of HPV infections
Source: PLoS Comput Biol. 2021 Sep 7;17(9):e1009352. doi: 10.1371/journal.pcbi.1009352 (PMC8448377; doi:10.1371/journal.pcbi.1009352)
Supplement: Attachment — Submitted filename: Reviewer_Comments_240521.pdf [file pcbi.1009352.s008.pdf]

Review for the manuscript entitled

Episome partitioning and symmetric cell divisions: quantifying the role of random events in the persistence of HPV infections

Manuscript Number: PCOMPBIOL-D-21-00650

Journal: PLOS Computational Biology

Summary:

In this manuscript by Beneteau et al., entitled "Episome partitioning and symmetric cell divisions: quantifying the role of random events in the persistence of HPV infections", the authors investigate the role of stochastic cellular events, in the persistence or extinction of HPV infections. They built a stochastic mathematical model of HPV dynamics combining intra- and inter-cellular stochastic processes. They show that the random partitioning of virus episomes at the intracellular level, and the symmetric divisions at the intercellular level, affect viral extinction.

Critique:

Methods:

Line 80, the term "asymmetric divisions" is used for the first time but is never defined in the manuscript. The term seems important for understanding this work and should already be defined in the introduction as is the term "symmetric divisions". Also, it is not clear how asymmetric divisions, is implemented in the model in comparison to symmetric ones, and how it affects HPV persistence.

Line 97, The authors mention the use of a Dirac or Poisson distribution for the intracellular episomes amplification. However, the rational for using one or/and the other distribution, is unclear. It would also be relevant to mention if they where already used in the literature for this purpose by citing other relevant studies.

Results, and supporting information:

Line 218: Fig S5. The legend for Figure S5 is provided but the figure is missing.

Also at line 388 – 390: "Concerning the choice of the intracellular multiplication regime (Dirac or Poisson distributed), we assign the Fixed multiplication regime when $pm_{i,j} < 0.5$ and the Poisson regime otherwise". It would be beneficial to state the biological relevance of this assignment of the distributions.

There is a Figure 5 provided for the main manuscript but no legend for it and no reference of it in the main manuscript.

There is a confusion in the manuscript and the supporting information between the matrices LHS_A, LHS_B, (line 142 – 150), LHS_1 (legend figure S5), LHS_2 (not mentioned in the manuscript but provided in the supporting information). How are they different from each other?

The findings and method in this study are really interesting. Yet, the technicalities and different conditions tested leading to the main findings are difficult to grasp. For this reason, this manuscript could benefit from a visual representation of the model. The intra- and intercellular dynamic could be represented as well as the different parameters / set of parameters / scenario considered.

Discussion:
In the discussion the authors wrote line 230 – 231: "Following this approach, we refine the model initially exposed by Ryser [19] and emphasize the risk of stochastic extinction." It should already be mentioned in the introduction, that the model is built on an already existing model that is now being improved.

Line 238: It is nicely stated that "symmetric divisions can also favour the spread of the virus in the tissue (when s > r).", however, this result is not shown in the manuscript. It would also be interesting to discuss in which molecular situation s > r and vice versa.

The authors discussed extensively the potential role of protein E1 in HPV persistence. How does this relate to the different HPV strains and their oncogenic potential? The authors should discuss if there are any intra- or inter-cellular molecular findings on the different oncogenic HPVs that could impact persistence as studied in their model.

Minor points:

Abstract:
In the Abstract, the use of the word Stochasticity is sometimes unprecise. In the sentence "Building on earlier work stressing the potential importance of stochasticity in HPV infection clearance", the authors should explicit which processes are referred to.

Methods:
Table 1: For the episome replication factor, the number of episome and the number of infected stem cells, a range (Min and Max) of the values used in this manuscript should be provided, in the table.

Results:
Figure 3: For the upper subplot, the legend explanation seems incomplete. It is not clear if there are one or two sets of dash lines to be considered. For these dash lines the colour coding should also be explained. And the legend should be changed in accordance to properly explain the graph.
For the lower subplot, some annotations in the graphs cannot be read properly.
