# Supplementary material for: Episome partitioning and symmetric cell divisions: Quantifying the role of random events in the persistence of HPV infections
Source: PLoS Comput Biol. 2021 Sep 7;17(9):e1009352. doi: 10.1371/journal.pcbi.1009352 (PMC8448377; doi:10.1371/journal.pcbi.1009352)
Supplement: Attachment — Submitted filename: response_reviewers.pdf [file pcbi.1009352.s009.pdf]

Response to reviewers *PLoS Comp Biol*

Thomas BENETEAU, Christian SELINGER,

Mircea T. SOFONEA, Samuel ALIZON

August 2021

Reply to the first reviewer's concerns

I carefully read the manuscript "Episome partitioning and symmetric cell divisions: quantifying the role of random events in the persistence of HPV infections", which develop a stochastic mathematical model of HPV dynamics with an explicit description of the intracellular level. This is a well organized and clearly written paper containing some valuable results. I have checked the code that underpins the findings, which is well commented and explained. In general, I suggest accepting the manuscript with minor revision.

Ans: We thank you for your positive appreciation!

However, there are some shortcomings that need to be addressed:

1. In the Author summary and the Introduction section, the logic of some sentences is not easy to understand, for example, sentences 4-5 in the author's summary: "Hence for a given HPV infection, the risk of progression towards cancerous status is low. Unfortunately, the prevalence of HPVs is very high, which makes it a major public health issue."

Ans: The sentence were indeed sometimes too dense. We changed their structure to clarify the Introduction.

2. The figures in the manuscript are a little oversized, but not of high quality, so they are not particularly clear.

   Ans: We made the changed and submitted vectorised files.

3. Why should the Acknowledgments be added between S6 and S7 instead of after S7?

   Ans: Thank you for pointing this mistake, which we corrected.

Second reviewer

Reviewer's comments on the paper entitled "Episome partitioning and symmetric cell divisions: quantifying the role of random events in the persistence of HPV infections" by Thomas et al.

This paper deals with the mathematical modelling of the Human Papillomaviruses (HPV), one of the most prevalent sexually transmitted infections (STI). The author developed a stochastic mathematical model of HPV dynamics with an explicit description of the intracellular level. They showed that the random partitioning of virus episomes upon stem cell division and the occurrence of symmetric divisions greatly affect viral persistence. Their findings gives a better understand the relative importance of stochasticity and immunity in HPV infection clearance. Numerical simulation is also performed to verify the analytical findings. This manuscript is written well and seems complete in every aspect.

Ans: We thank the reviewer for this positive and accurate summary.

However, the reviewer feels that the authors should discussed some recent studies

Ans: We thank the reviewer for her/his suggestions, which we read carefully when we could

access them. However, for some of the references put forward, we had troubles finding the relationship with the contents of our manuscript. For instance, some references were using different methods for different viruses. We discuss each reference in details and would be happy to add all of them if the reviewer wishes to point explain his/her motivation for adding them.

1. Chatterjee, A. N, Ahmad, B., "A fractional-order differential equation model of COVID-19 infection of epithelial cells", Chaos, Solitons & Fractals , V 147 (2021), 110952.

   Ans: This is a model using ODE to study COVID-19 dynamics so we fail to see the relationship with our study.

2. Mondal, Jayanta, Piu Samui, and Amar Nath Chatterjee. "Optimal control strategies of non-pharmaceutical and pharmaceutical interventions for COVID-19 control." Journal of Interdisciplinary Mathematics (2020): 1-29.

   Ans: This is an optimal control model to study COVID-19 dynamics so we fail to see the relationship with our study. (Incidentally, we developed a similar optimal control model in the team, which was published in *PLoS Computation Biology* by Richard *et al.*)

3. Chatterjee, Amar Nath, Fahad Al Basir, and Yasuhiro Takeuchi. "Effect of DAA therapy in hepatitis C treatment—an impulsive control approach." Mathematical Biosciences and Engineering 18, no. 2 (2021): 1450-1464.

   Ans: This is a model using impulsive control approach to study HCV treatment. It is a within-host dynamics model so more related to ours but neither the methods not the biology fits.

4. Chatterjee, A. N. and Al Basir, F., 2020. A model for sars-cov-2 infection with treatment. Computational and mathematical methods in medicine, 2020.

Ans:

5. Chakraborty, Sudip, and Priti Kumar Roy. "Therapeutic control of HPV associated cervical cancerous cell and its possible extinction." Nonlinear Studies 26, no. 2 (2019).

   Ans: We did not have access to this paper unfortunately. Based on the title, there seems to be little links with our study since it focuses on cancer dynamics and therapeutics. Our study is more on viral dynamics in non-persisting infections

6. Chakraborty, S., Xianbing Cao, S. Bhattyacharya, and P. K. Roy. "The Role of HPV on cervical cancer with several functional response: a control based comparative study." Computational Mathematics and Modeling 30, no. 4 (2019): 439-453.

   Ans: This is also a study on cancer dynamics, so, from a biological standpoint, the link with our study is weak. From a mathematical point of view, the authors use an optimal control approach on an ODE based model. Again, since we use branching processes and stochastic modelling, we are unsure how relevant this study is to ours. However, it is true that there are relatively few within-host models of HPV. To stress this point without citing a myriad of articles, we now refer to the review article by Ryser et al (2017, *Papillomavirus Research*, 3:46–49) which lists many mechanistic models of HPV dynamics.

7. Chakraborty, Sudip, Xue-Zhi Li, and Priti Kumar Roy. "How can HPV-induced cervical cancer be controlled by a combination of drug therapy? A mathematical study." International Journal of Biomathematics 12, no. 06 (2019): 1950070.

   Ans: The same comments are true for this article: the mathematical approach is very different and the biological link is tenuous. As indicated above, we now refer to the review article by Ryser et al (2017) to stress the paucity of HPV modelling.

The overall manuscript seems fine and it may be accepted if authors incorporate the above points/query in the revised manuscript.

Third reviewer

Review for the manuscript entitled Episome partitioning and symmetric cell divisions: quantifying the role of random events in the persistence of HPV infections Manuscript Number: PCOMPBIOL-D-21-00650 Journal: PLOS Computational Biology

Summary:

In this manuscript by Beneteau et al., entitled "Episome partitioning and symmetric cell divisions: quantifying the role of random events in the persistence of HPV infections", the authors investigate the role of stochastic cellular events, in the persistence or extinction of HPV infections. They built a stochastic mathematical model of HPV dynamics combining intra- and inter-cellular stochastic processes. They show that the random partitioning of virus episomes at the intracellular level, and the symmetric divisions at the intercellular level, affect viral extinction.

Ans: Thank you for this accurate summary.

Critique:

Methods:

- Line 80, the term "asymmetric divisions" is used for the first time but is never defined in the manuscript. The term seems important for understanding this work and should already be defined in the introduction as is the term "symmetric divisions". Also, it is not clear how

asymmetric divisions, is implemented in the model in comparison to symmetric ones, and how it affects HPV persistence.

Ans: Thank you for stress this! We now define this term in the Introduction.

- Line 97, The authors mention the use of a Dirac or Poisson distribution for the intracellular episomes amplification. However, the rational for using one or/and the other distribution, is unclear. It would also be relevant to mention if they where already used in the literature for this purpose by citing other relevant studies.

  Ans: We now clarify this choice, which was largely driven by the fact that Dirac and Poisson distributions are the most parsimonious, hence common, distributions one can choose when dealing with a random count of entities. The Dirac distribution corresponds to the extreme case when the focal number is not random at all (but a fixed scalar parameter), while the Poisson distribution implies that the focal number is the result of a large number of identical and independent of trials, each one having a low probability of success.

Results, and supporting information:

- Line 218: Fig S5. The legend for Figure S5 is provided but the figure is missing.

  Ans: We apologise for this oversight. We have added the figure.

- Also at line 388 – 390: "Concerning the choice of the intracellular multiplication regime (Dirac or Poisson distributed), we assign the Fixed multiplication regime when pm i,j $<$ 0.5 and the Poisson regime otherwise". It would be beneficial to state the biological relevance of this assignment of the distributions.

  Ans: As explained above, the choice of the Dirac and Poisson distributions are motivated by their being the two most parsimonious ways to model a count process, as they only require

one parameter to be specified. This is now stressed in the text

There is a Figure 5 provided for the main manuscript but no legend for it and no reference of it in the main manuscript.

Ans: Thank you, we corrected this error and included the missing figure.

There is a confusion in the manuscript and the supporting information between the matrices LHS_A, LHS_B, (line 142 – 150), LHS_1 (legend figure S5), LHS_2 (not mentioned in the manuscript but provided in the supporting information). How are they different from each other?

Ans: Thank you for pointing out this typo! LHS_1 was equivalent to LHS_A.

The findings and method in this study are really interesting. Yet, the technicalities and different conditions tested leading to the main findings are difficult to grasp. For this reason, this manuscript could benefit from a visual representation of the model. The intra- and intercellular dynamic could be represented as well as the different parameters / set of parameters / scenario considered.

Ans: Thank you! We now provide an extra figure in the supplementary materials that graphically summarise the different aspects of the model.

Discussion:

In the discussion the authors wrote line 230 – 231: "Following this approach, we refine the model initially exposed by Ryser [19] and emphasize the risk of stochastic extinction." It should already be mentioned in the introduction, that the model is built on an already existing model that is now being improved.

Ans: We clarified the Introduction so that there is no ambiguity that we are building on the

model by Ryser et al.

Line 238: It is nicely stated that “symmetric divisions can also favour the spread of the virus in the tissue (when s > r).”, however, this result is not shown in the manuscript. It would also be interesting to discuss in which molecular situation s > r and vice versa.

Ans: We show in the S4 section of the supplementary materials that the extinction probability of the intercellular process is equal to $min(1, r/s)$. Hence, the probability of extinction decrease with increasing values of s when $s > r$

The authors discussed extensively the potential role of protein E1 in HPV persistence. How does this relate to the different HPV strains and their oncogenic potential? The authors should discuss if there are any intra- or inter-cellular molecular findings on the different oncogenic HPVs that could impact persistence as studied in their model.

Ans: Our model revolves around the early stages of infection. It would be interesting to discuss in more details how our results relate to differences between genotypes but very little is known with that aspect in acute infection as discussed in Alizon et al (2017, Viruses). To take a single example, it is even unclear that infections caused by HPV16, the most oncogenic genotype, are associated with higher virus loads. Furthermore, more than 90% of the infections we study will not become chronic (Insinga et al 2007) and even less cause cancer. Furthermore, in addition to the chronicity itself, oncogenicity involves many molecular processes inferring with the cellular machinery and immune recognition (Doorbar et al 2012 Vaccine) that are not included in our model. Overall, although the symmetric division has seducing parallels with oncogenic cell divisions, our message is more that care should be taken before equating the two.

Minor points:

Abstract:

In the Abstract, the use of the word Stochasticity is sometimes unprecise. In the sentence "Building on earlier work stressing the potential importance of stochasticity in HPV infection clearance", the authors should explicit which processes are referred to.

Ans: We clarified what we mean by stochasticity.

Methods:

Table 1: For the episome replication factor, the number of episome and the number of infected stem cells, a range (Min and Max) of the values used in this manuscript should be provided, in the table.

Ans: We now specify the constraints associated with each quantity.

Results:

Figure 3: For the upper subplot, the legend explanation seems incomplete. It is not clear if there are one or two sets of dash lines to be considered. For these dash lines the colour coding should also be explained. And the legend should be changed in accordance to properly explain the graph. For the lower subplot, some annotations in the graphs cannot be read properly.

Ans: We updated figure 3 following your recommendations.
